# Icariin regulates stem cell migration for endogenous repair of intervertebral disc degeneration by increasing the expression of chemotactic cytokines

**DOI:** 10.1186/s12906-022-03544-x

**Published:** 2022-03-10

**Authors:** Zhaofei Zhang, Fengwei Qin, Yonghui Feng, Sineng Zhang, Chunliang Xie, He Huang, Chaohui Sang, Shaoyu Hu, Feng Jiao, Jie Jiang, Yi Qin

**Affiliations:** 1 Department of Spine and Orthopedics, Zhuhai People’s Hospital (Zhuhai Hospital Affiliated With Jinan University), Zhuhai, 519000 Guangdong People’s Republic of China; 2 Department of Orthopedic Surgery, Guangzhou Hospital of Integrated Traditional and Western Medicine, 87 Yingbin Road, Huadu District, Guangzhou, Guangdong People’s Republic of China

**Keywords:** Icariin, Nucleus pulposus, Intervertebral disc degeneration, Polysaccharides

## Abstract

**Background:**

Icariin (ICA) can promote the migration and bone formation of bone marrow mesenchymal stem cells. This study explored a potential role of ICA in recruiting stem cell niches (SCNs) within the intervertebral disc region (ISN)-derived stem cells (ISN-SCs) to treat intervertebral disc degeneration (IVDD).

**Materials and methods:**

EdU staining, transwell, and wound healing tests were used to analyze the function of ICA on ISN-SCs proliferation and migration ability. Simultaneously, the IVDD rat model was constructed by the acupuncture and divided into Sham, Sham + ICA, IVDD, and IVDD + ICA groups. H&E and PAS staining were performed to detect the pathological changes of IVDD tissues. Immunofluorescence was performed to discover relevant marker expression on the surface of stem cells in the IVDD tissues. Western blot and qPCR were executed to find the protein and mRNA expression of related cytokines in the IVDD tissues.

**Results:**

ISN-SCs treated with 1 μM ICA obtained the better ability of proliferation and migration. H&E staining showed that the annulus fibrosus in the IVDD group was obviously hyperplasia with cavities and fissures; the nucleus pulposus was reduced. PAS staining showed that the content of polysaccharides was significantly reduced in the nucleus pulposus of IVDD group. However, the ICA treatment alleviated the pathological trends of the IVDD tissues. Simultaneously, ICA treatment increased significantly the expression of stem cells and IGF-1, TGF-β, SDF-1, CCL-5, Collagen I, Collagen II, Aggrecan, and SOX9 in IVDD tissues.

**Conclusions:**

ICA treatment promoted the migration of stem cell in IVDD by increasing the expression of chemotactic cytokines, including IGF-1, TGF-β, SDF-1, and CCL-5.

**Supplementary Information:**

The online version contains supplementary material available at 10.1186/s12906-022-03544-x.

## Background

Low back pain is one of the most common symptoms of orthopedic illness and a common public health problem related to lumbar intervertebral disc (IVD) degeneration (IVDD) [1, 2]. Over 80% of adults will indicate back pain in their lives, which is the most common cause of restricted mobility in people under 45 [3]. IVDD can be clinically manifested as spinal stenosis, axial back pain, radiculopathy, or myelopathy [4]. Severe IVDD not only disables the patient’s ability to work and affects the quality of life, but also brings a huge economic burden to society [5]. Therefore, it is very important to find an effective treatment for IVDD.

IVD in healthy people has a complex structure, including three different parts: central nucleus pulposus (NP); cartilaginous endplate (EP); and fibrocartilaginous annulus fibrous (AF) tissue and its inner and outer regions, consisted of concentric fibrous tissue layers [4]. Among them, AF contains abundant parallel collagen fibers, NP includes a gel-like matrix composed of abundant proteoglycan aggrecan and little collagen and elastin fibers, and EP contains a hyaline cartilage and round cartilage cells. These structures help the spine to restore its original shape after bending or stretching [4]. When IVDD occurs, the synthesis of glycosaminoglycan, proteoglycan, collagen II is reduced, but acidic pH and catabolic proteinases increase, resulting in the gradual loss of disc components and cell density [6]. Current treatments for IVDD include the administration of pain medications or surgical procedures, however, these methods have not achieved the desired therapeutic effect [7].

After understanding the internal mechanism of stem cell movement and migration, activating endogenous stem cells to restore and rebuild the IVDD is considered to be a better biological treatment and can avoid the trauma caused by surgical treatment *in vivo* [8]. Studies have shown that degenerated IVD tissue can recruit stem cells through the release of chemotactic cytokines, including transforming growth factor (TGF)-β, stromal cell-derived factor-1 (SDF-1), insulin-like growth factor 1 (IGF-1), chemokine C–C motif ligand (CCL) 5 [9–12]. Moreover, research has shown that icariin (ICA), is a flavonoid extract of several genus Epimedium species, prevents neuroinflammation and weakens oxidative stress damage [13], which shows the ability to enhance the bones and muscles, and can effectively enhance bone marrow mesenchymal stem cell (BMSC) osteogenesis and proliferation [14, 15]. ICA can also enhance the proliferation and of growth neural stem cells by regulating the expression of cell cycle genes and proteins [16]. In addition, ICA regulate gene expression to promote the proliferation of human neural stem cells, showing potential neuroprotective effect on nerve [17]. ICA also increase the function and of viability human NP-derived mesenchymal stem cells [18]. As shown above, ICA can promote the vitality and proliferation of various stem cells. Other studies have shown ICA has the protective function on human NP cells in IVDD through the nuclear factor erythroid 2-related factor 2 (Nrf-2) signaling [19]. ICA attenuates inflammatory response of human NP cells induced by interleukin-1β [20]. In addition, ICA can highly express TGF-β in BMSCs [21] and IGF-1 in mouse dermal papillary cells [22]. Therefore, this study hypothesizes that ICA may induce the migration of stem cells to repair degenerated IVD by promoting the expression of cytokines.

Recently, a potential stem cell niches within the IVD region (ISN) have been reported to play an important role in IVD regeneration [23]. In this study, ICA was used to study the role on the migration and proliferation of ISN-SCs cells. Western blot and qPCR were executed to analyze the protein and mRNA expression of related cytokines in the IVDD tissues treated with ICA to explore the potential mechanism for endogenous repair of IVDD and to offer a research basis for ICA in treating IVDD.

## Methods

### Isolation and identification of ISN-SCs

ISN-SCs were separated from 3-month-old male Wister rats (320–380 g, Guangdong Experimental Animal Center) according to the following procedure. The rats were euthanized after intraperitoneal injection of sodium pentobarbital (150 mg/kg). The functional spinal units of L8–L9 were isolated in the Wister rats. Then tissues of the ISN regions were isolated using the scalpel under sterile conditions [23] and were then digested by using 0.15% collagenase II in DMEM/F12 (1:1; Gibco, USA) including 3% fetal bovine serum (FBS) at 37 °C for 6 h. Before centrifuging for 5 min at 1000 rpm, the suspension was then filtered using a 70-μm cell strainer. The cell pellet was resuspended using DMEM/F12 added with 1% penicillin–streptomycin and 10% FBS and maintained in an incubator with 5% CO_2_ at 37 °C. When the ISN-SCs cultured to passage 4 (P4) for 24 h, cells were stained with CD29, CD90, CD44, CD45 and CD11b antibodies for 2 h and identified by the flow cytometry (BD, USA).

### CCK-8 assay for cell viability

P4 ISN-SCs were plated in a 96-well plate with a density of 1 × 10^3^ cells/well for reaching 60% confluency. Then, serum-free DMEM/F12 containing 0.1 μM, 1 μM, and 10 μM ICA were used to incubate cells. After incubation with ICA for 24, 48, and 72 h, CCK8 (7Sea Biotech, China) assay was executed to detect cell viability at 450 nm based on the protocol using the microplate reader (TECAN, Switzerland).

### EdU staining for cell proliferation

P4 ISN-SCs were laid in a 24-well plate with a density of 3 × 10^3^ cells/well for 24 h, incubated with serum-free DMEM/F12 containing different concentrations of ICA for 48 h, and treated with serum-free DMEM/F12 comprising 10 μmol/L EdU (RiboBio, China) for 2 h. The treated cells were fixed by 4% paraformaldehyde and stained by DAPI for 5 min. Cells were surveyed using a fluorescence microscope to evaluate the cell proliferation ability.

### Cell migration ability

According to the manufacturer’s procedures, the six-well transwell chambers (Millipore) were used to study the cell migration. Briefly, 1 × 10^5^ P4 ISN-SCs from serum-free DMEM/F12 were laid in the upper transwell chamber of insert for 24 h and treated with different concentrations of ICA, and 500 μL of DMEM/F12 supplemented with 10% FBS was added into the lower transwell chamber. The P4 ISN-SCs were cultured for 48 h. Then, P4 ISN-SCs were fixed in 100% methanol for 30 min and stained with 0.5% crystal violet (Sigma-Aldrich, USA) for 20 min. The migrating cells were observed under the fluorescence microscope. Simultaneously, P4 ISN-SCs were seeded in 24-well plates and P4 ISN-SCs layer was scratched using the tip of a 200 μL pipette. The wound width was observed for 48 h after the cells incubated with different concentrations of ICA to assess the healing ability of detected P4 ISN-SCs.

### Phalloidin staining for filamentous actin

P4 ISN-SCs were cultured on coverslips and treated with different concentrations of ICA for 48 h. P4 ISN-SCs were fixed with 100% methanol for 30 min. Then, P4 ISN-SCs were permeabilized with 0.3% of Triton in PBS for 5 min and treated with 1% of bovine serum albumin (BSA, Sigma-Aldrich) in PBS for 30 min. Phalloidin-FITC (Sigma-Aldrich) together with DAPI (Sigma-Aldrich) was used for incubating P4 ISN-SCs for 1 h. Images were obtained using a confocal microscope (Zeiss, Germany) to evaluate the expression of filamentous actin (F-actin) in P4 ISN-SCs.

### Construction and treatment of IVDD rat model

3 month-old Wister rats 320–380 g were selected to build rat model. The IVDD model was established by puncturing the 8th and 9th vertebrae with a needle of defined gauge with outer diameter of 0.45 mm. The needle was punctured from the skin to a depth of 6 mm through the NP. Before extraction, the needle was rotated 360° and stayed in place for 1 min. IVDD rat model was divided into Sham, Sham + ICA, IVDD, and IVDD + ICA groups and 12 rats in each group. When IVDD rats were intraperitoneally injected with ICA for 4 weeks and 8 weeks, they were used for subsequent experimental analysis.

### Histopathological observation

Rats were killed to observe the pathological changes in the IVD of Sham, Sham + ICA, IVDD, and IVDD + ICA groups. The L8-9 IVD together with the adjacent bilateral half of the vertebral bodies in rats were harvested and fixed in 10% neutral buffered formalin for 48 h. 10% EDTA solution was used to decalcify for the IVD for three weeks and then paraffin was used to embed the tissue. Sections collected from the IVD samples embedded were stained with H&E and PAS staining and observed by microscope.

### Immunofluorescence

Paraffin-embedded IVD tissues were dewaxed, and then antigen retrieval was performed. The slides were sealed using 1% BSA at 37 °C for 60 min; and incubated with primary antibodies (CD90: red; CD105: green). at room temperature for 2 h and followed by secondary antibodies at 37 °C for 90 min; and were dyed in DAPI for 5 min. All sections were sealed with cover glass and viewed using a confocal microscope (Zeiss, Germany).

### qPCR

TRIzol reagent (Invitrogen, USA) was used to extract the total RNA of IVD tissues. qPCR was used to detected the mRNA expression. Reactions were executed in 50-µL volumes comprising SYBR Green PCR master mix through a GeneAmp PCR System 9600. Thermal cycling conditions was 40 cycles at a temperature of 95 °C for 30 s, a temperature of 60 °C for 30 s, and 72 °C for 2 min. Data were collected and analyzed. The mRNA expression was calculated by 2^−ΔΔCT^ method. The primer sequences used in this study were listed in Table 1.

**Table 1:** The sequences of primers that were used in this study

Gene	Sequence(5’- 3’)	Product Length(bp)
GAPDH F	CCTCGTCTCATAGACAAGATGGT	169
GAPDH R	GGGTAGAGTCATACTGGAACATG	
CCL-5 F	CTCCCCATATGGCTCGGAC	85
CCL-5 R	AAAATACTCCTTCACGTGGGC	
IGF-1 F	CCGGGACGTACCAAAATGAG	96
IGF-1 R	GGTAAAGGTGAGCAAGCAGA	
SDF-1 F	TCTTTGAGAGCCATGTCGCC	86
SDF-1 R	CCTTGCAACAATCTGAAGGGC	
SOX9 F	GCAACAGATCTCCTACAGCC	86
SOX9 R	CGGTGTAGTCATACTGCGAG	
TGF-B F	GCAACAATTCCTGGCGTTAC	136
TGF-B R	CTGAAGCGAAAGCCCTGTAT	
Collagen I-F	GAGCCAGCAGATTGAGAAC	127
Collagen I-R	TTGGTTAGGGTCGATCCAGT	
Collagen II-F	GCCAGGATGCCCGAAAATTA	288
Collagen II-R	CGTCAAATCCTCCAGCCATC	
Aggrecan-F	GCAATTTGAGAAGTGGCGTC	115
Aggrecan-R	GTAATTGCAGGGGACATCGT	

### Western blot

The RIPA buffer (Solarbio Life Sciences, China) was used to extract the total protein of samples from IVD tissues, according to the protocol. The BCA Protein Assay Kit (Thermo Fisher Scientific) was used to measure protein contant. Approximately 25 μg of total protein was boiled, separated using 12% SDS-PAGE, and blotted onto PVDF membranes. Then the membranes were sealed with 5% skimmed milk for 2 h, incubated with primary antibodies against IGF-1 (1:1000, ab223567, Abcam, Cambridge, MA, USA), TGF-β (1:500, ab92486, Abcam), SDF-1 (1:1000, ab18919, Abcam), CCL-5 (1:1000, ab189841, Abcam), SOX9 (1:1000, ab26414, Abcam), Aggrecan (1:1000, ab3778, Abcam), Collagen I (1:1000, ab233080, Abcam), Collagen II (1:1000, ab188570, Abcam) and GAPDH (1:5000, ab22555, Abcam), followed by incubation with horseradish peroxidase-conjugated secondary antibody for 2 h at room temperature. Blots were visualized using a chemiluminescence reagent (Millipore, USA). GAPDH was the internal reference. The relative expressions of proteins were normalized to GAPDH using Image-Pro software. The antibody information used in this study were as follows.Western blot experimental results of the original film in the supplementary file 1.

### Statistics

SPSS V16.0 software (IBM, USA) was used for statistical analyses. Differences among multiple groups were evaluated using one-way analysis of variance, followed by Dunnett’s test or Tukey’s test. *P* < 0.05 was considered a significant difference.

## Results

### Identification of ISN-SCs and the effect of ICA on ISN-SCs proliferation

**Fig. 1:**
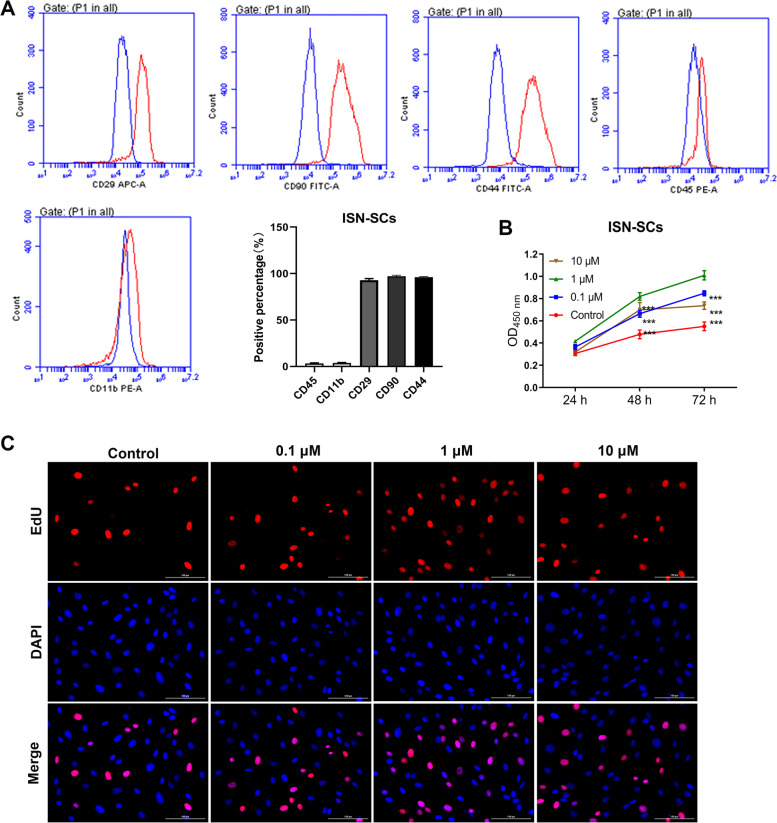
The effect of ICA on proliferation of SCNs within the ISN-SCs.** A** CD29, CD90, CD44, CD45, and CD11b expression levels on ISN-SCs were detected by a flow cytometry. **B** The absorbance of ISN-SCs treated with different concentration of ICA for different time was detected at 450 nm by CCK-8 assay. Differences among multiple groups were evaluated using one-way analysis of variance, followed by Dunnett’s test. *** *P* < 0.001 vs. Control. **C** The proliferation ability of ISN-SCs treated with different concentration of ICA was determined by EdU staining

To study the role of ICA in regulating stem cell migration for endogenous repair of IVDD, ISN-SCs were separated from 3-month-old male Wister rats. The flow cytometry was used to analyze the expression of P4 ISN-SCs surface antigens (Fig. 1A). The results showed that the positive expression of ISN-SCs positive markers, including CD29 (92.83%), CD90 (97.10%), and CD44 (96.10%), was about 100%, and the positive expression of ISN-SCs negative markers CD45 (3.34%) and CD11b (3.74%) was extremely weak, suggesting that ISC-SCs were successfully isolated (Fig. 1A). Regarding the function of different concentrations of ICA on the proliferation ability of ISN-SCs, the CCK8 assay showed that the proliferation abilities of ISC-SCs treated with 0.1 μM and 10 μM ICA for 48 h were significantly higher than those of ISC-SCs untreated with ICA for 48 h (Fig. 1B). In addition, compared to ISC-SCs treated with 0.1 μM or 10 μM ICA, ISC-SCs treated with 1 μM ICA obtained highest proliferation ability, and as time increased to 72 h, cell viability gradually increased (Fig. 1B). Simultaneously, EdU staining results also showed that ISC-SCs treated with 1 μM obtained highest proliferation ability (Fig. 1C). The above results indicated that 1 μM ICA had a better ability to promote cell proliferation.

### The effect of ICA on ISN-SCs migration and cytoskeleton formation

**Fig. 2:**
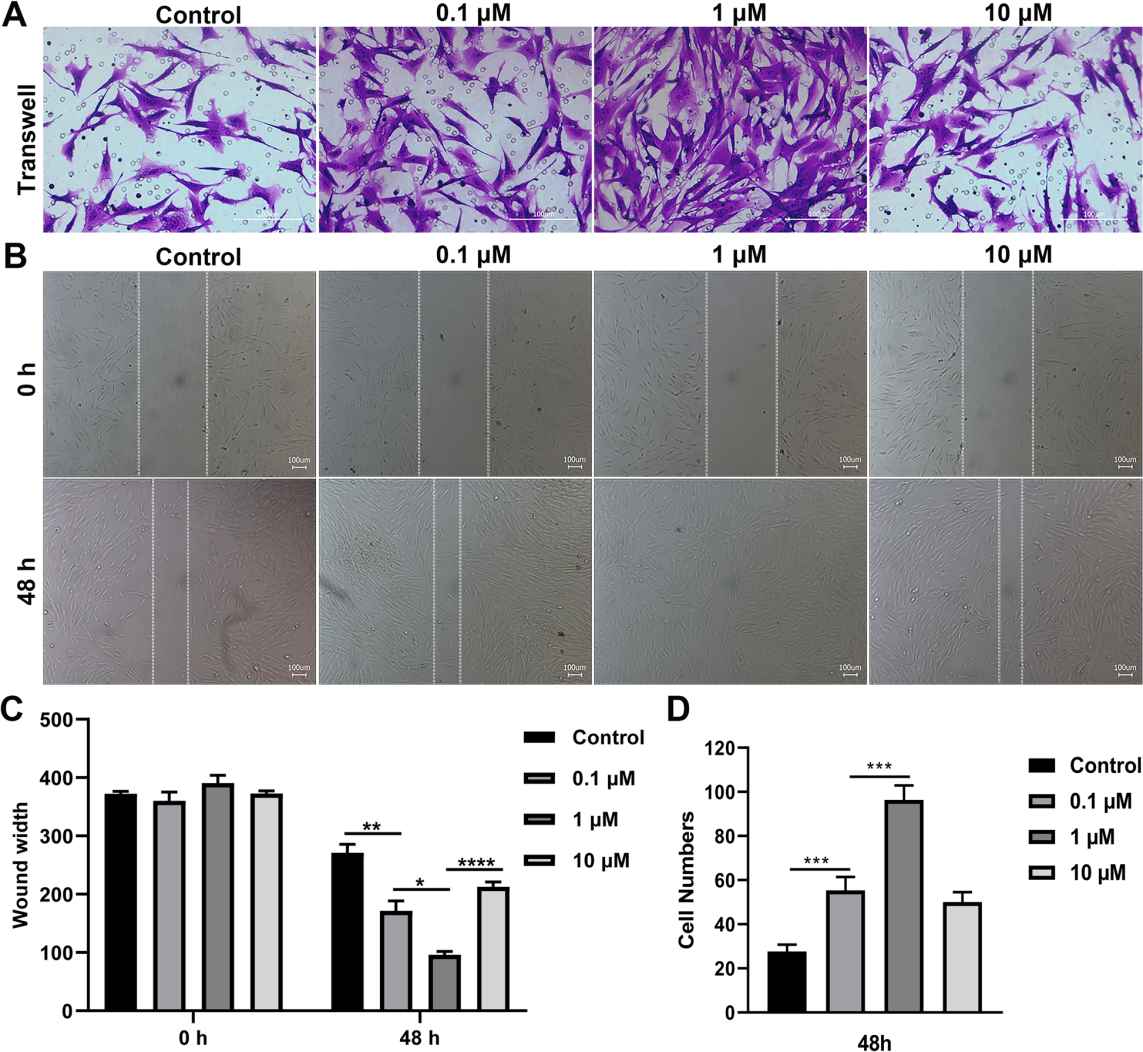
The effect of ICA on migration of SCNs within the ISN-SCs. **A** The migration ability of ISN-SCs treated with different concentration of ICA was estimated by transwell assay. **B** The healing ability of ISN-SCs treated with different concentration of ICA was estimated by wound healing for 48 h. **C** Semi quantitative analysis of healing ability of graph B. **D** Semi quantitative analysis of migration ability of graph A. Differences among multiple groups were evaluated using one-way analysis of variance, followed by Tukey’s test. * *P* < 0.05, ** *P* < 0.01, *** *P* < 0.001, and **** *P* < 0.0001

**Fig. 3:**
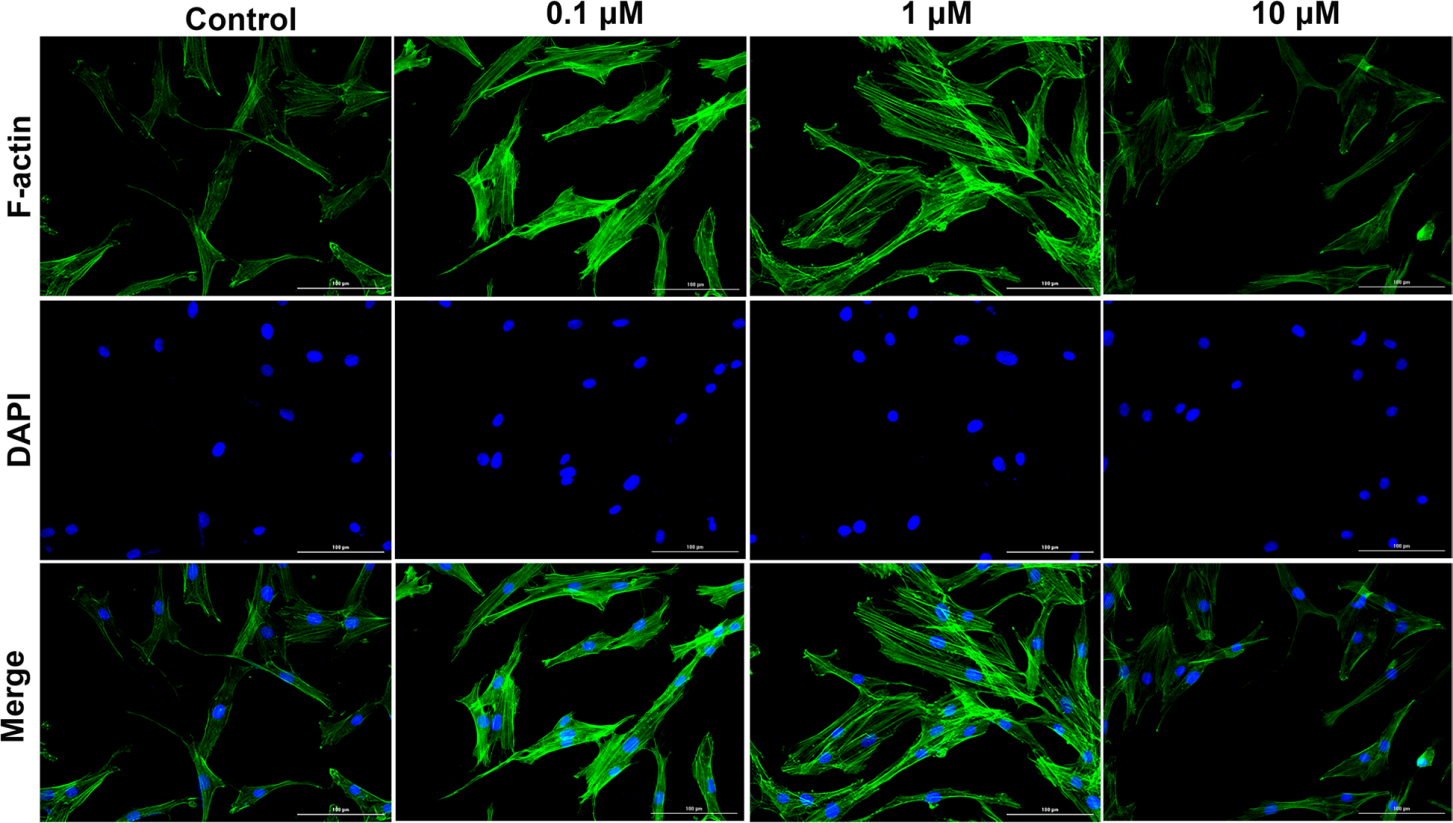
The effect of ICA on bone formation of SCNs within the ISN-SCs

Based on the above research, we further studied the function of different concentrations of ICA on cell migration and skeletal formation. The transwell results indicated that the migration abilities of ISC-SCs treated with 0.1 μM and 10 μM ICA for 48 h were higher than those of ISC-SCs untreated with ICA for 48 h, moreover, ISC-SCs treated with 1 μM ICA obtained highest migration ability (Figs. 2A and D). The wound healing results showed that these cells incubated with 0.1 μM and 10 μM ICA were higher migratory than scrambled control at 48 h after scratching, more importantly, 1 μM ICA distinctively increased the cell migration of ISC-SCs (Figs. 2B and C). In addition, phalloidin staining results showed that ISC-SCs incubated with 1 μM ICA obtained the highest expression of F-actin (Fig. 3). These results implied that 1 μM ICA gained a better ability to promote cell migration and skeleton formation.

### Pathological changes of IVDD tissue after ICA treatment

**Fig. 4:**
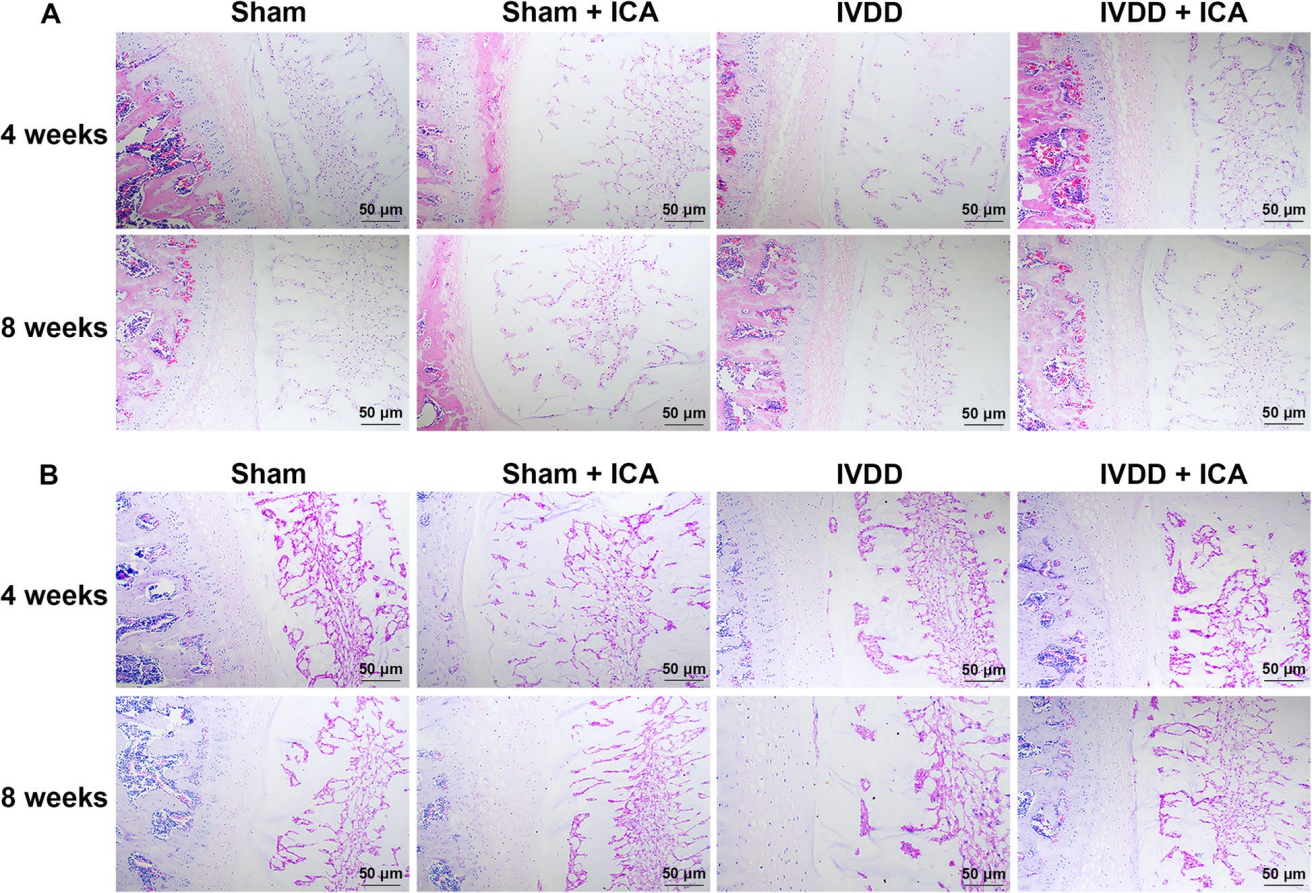
H&E (**A**) and PAS (**B**) staining were used to detect the effect of ICA on the pathological changes of intervertebral disc tissue

After in vitro cell experiments proved that ICA has a good ability to promote cell proliferation and migration, *in vivo* animal experiments were used to explore the therapeutic effect of ICA on IVDD through H&E and PAS staining. H&E staining showed that compared to Sham group, the AF in the IVDD group was obviously hyperplasia with cavities and fissures and the NP was reduced 4 weeks after the construction of IVDD rat model (Fig. 4A). PAS staining results showed that compared to Sham group, the content of polysaccharides was significantly reduced in the NP of IVDD group 4 weeks after the construction of IVDD rat model (Fig. 4B). However, the ICA treatment for 4 weeks alleviated the pathological phenomenon of the IVDD tissues, and with the treatment time extending to 8 weeks, the pathological changes of the IVDD tissue were significantly improved (Figs. 4A and B).

### The potential mechanism of ICA on stem cell recruitment and treatment of IVDD

**Fig. 5:**
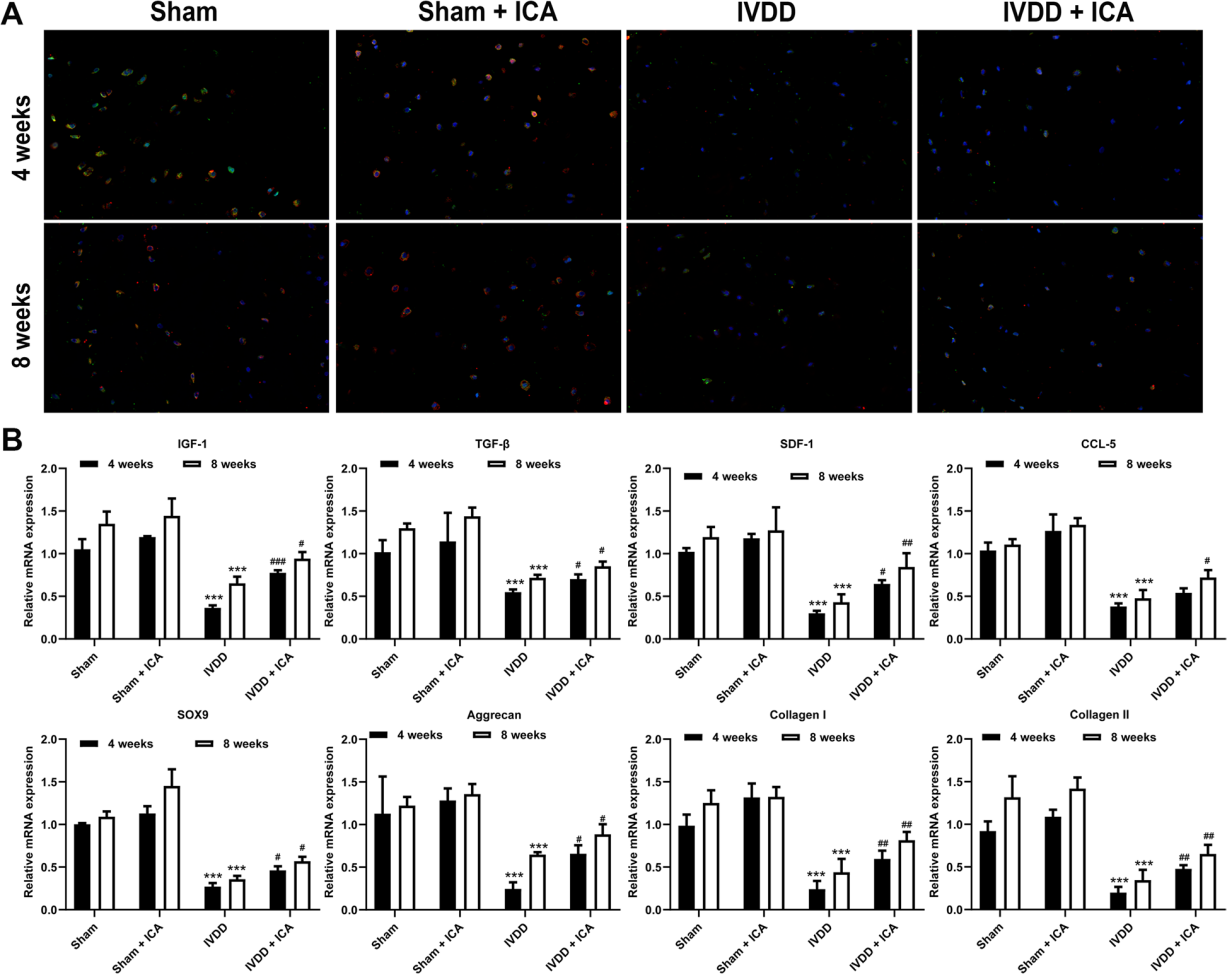
The effect of ICA on the expression level of intervertebral disc stem cells (**A**) and mRNA (**B**) was detected by immunofluorescence and qPCR. CD90: red; CD105: green. Differences among multiple groups were evaluated using one-way analysis of variance, followed by Tukey’s test. *** *P* < 0.001 vs. Sham; and ^#^ *P* < 0.05, ^##^ *P* < 0.01, and ^###^ *P* < 0.001 vs. Sham + ICA

**Fig. 6:**
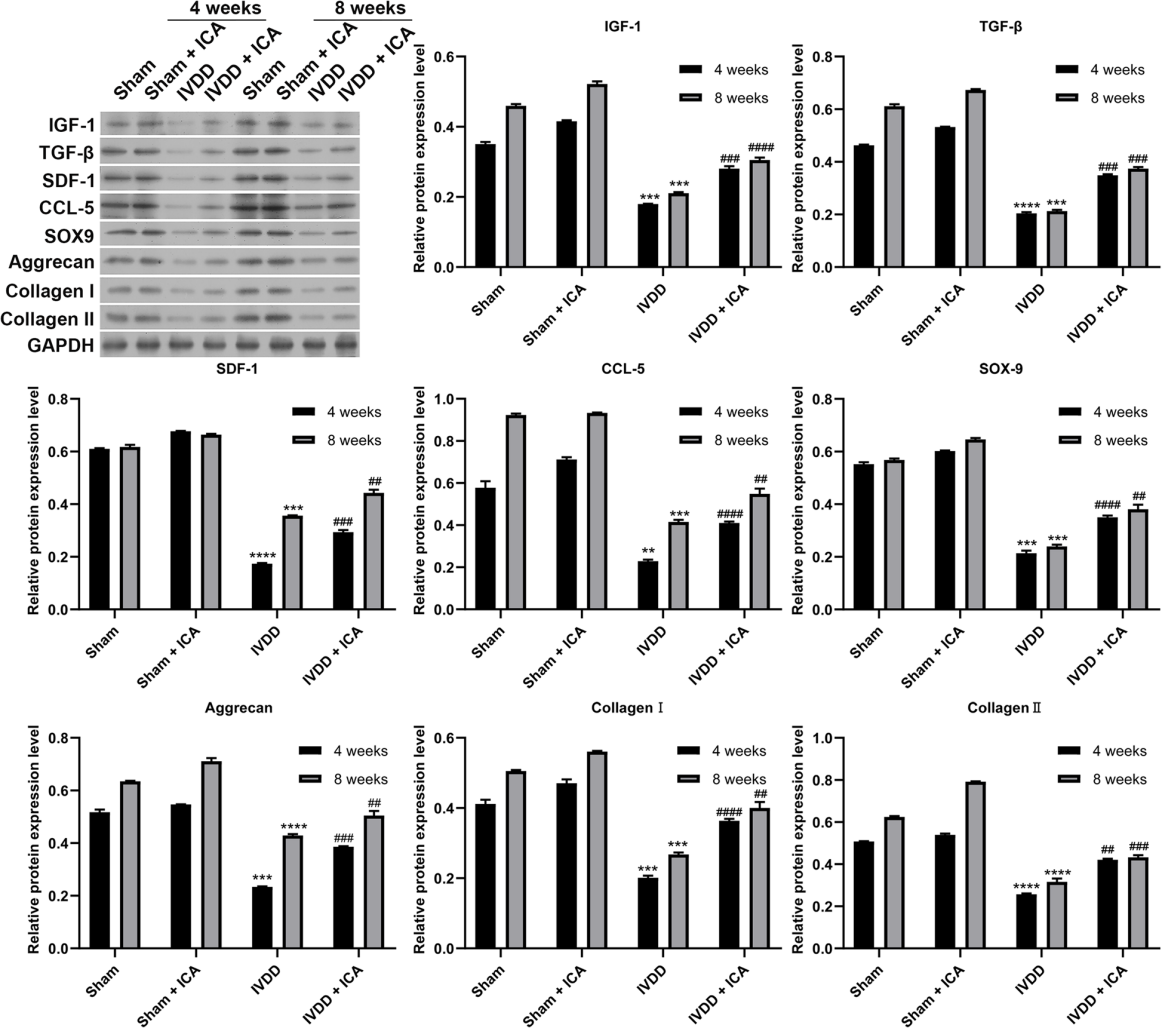
The effect of ICA on the protein expression level of intervertebral disc tissue was detected by western blot. Differences among multiple groups were evaluated using one-way analysis of variance, followed by Tukey’s test. ** *P* < 0.01, *** *P* < 0.001, and **** *P* < 0.0001 vs. Sham; and ^##^ *P* < 0.01, ^###^ *P* < 0.001, and ^####^ *P* < 0.0001 vs. Sham + ICA

In this study, the potential mechanism of ICA to treat IVDD in rats was further studied. The immunofluorescence results showed that, for 4 weeks of ICA treatment, green and red fluorescence in IVD tissues of Sham + ICA group were stronger than those of Sham group and were no significant change compared to ICA applied to treated rats with IVDD for 8 weeks (Fig. 5A). And the green and red fluorescence in IVD tissues of IVDD were weaker than those of Sham group (Fig. 5A). In addition, for 4 weeks of ICA treatment, the green and red fluorescence in IVD tissues of IVDD + ICA group were stronger than those of IVDD group and were weaker than those in IVDD + ICA group with ICA applied to treated rats with IVDD for 8 weeks (Fig. 5A). Moreover, the protein and mRNA expression levels of IGF-1, TGF-β, SDF-1, CCL-5, SOX9, Aggrecan, Collagen I, and Collagen II in IVDD tissues of Sham group and Sham + ICA group 4 weeks after IVDD rat model were lower than those of Sham group and Sham + ICA group 8 weeks after IVDD rat model, and significantly higher than those of IVDD group 4 weeks after IVDD rat model (Figs. 5B and 6). Conversely, ICA treatment could greatly reverse the protein and mRNA expression of most related factors, including IGF-1, TGF-β, SDF-1, SOX9, Aggrecan, Collagen I, and Collagen II, in IVD tissues of IVDD group, and with ICA treatment to 8 weeks, the expression level of these factors gradually increased, suggesting that ICA might promote the repair of ISN-SCs in IVDD by increasing the expression of chemotactic cytokines, including IGF-1, TGF-β, SDF-1, and CCL-5 (Figs. 5B and 6).

## Discussion

Since the conception of SCNs was proposed [24], niches in various organs and tissues have been observed, containing the nervous, digestive, and respiratory systems, along with heart, bone marrow, skin, placental, and adipose tissues [25]. SCNs is consisted of extracellular matrix and other adjacent cells, which is a dynamic microenvironment that can balance the activities of stem cells to maintain tissue homeostasis and repair during the entire life cycle of organisms [25, 26]. The migration of niche endogenous stem cells to adjacent target areas is a key process of tissue self-healing. A potential SCNs, which is perichondrium region adjacent to the EP and outer zone of the AF, is shown the great significance in IVD regeneration processes [23, 27]. In this study, ISN-SCs were isolated and treated with 1 μM ICA obtained the better proliferation and migration. ICA treatment showed that obvious hyperplasia with cavities and fissures in the AF in the IVDD group and decreased polysaccharides in the NF of IVDD group were relieved and increased the expression level of stem cells in the IVDD tissues *in vivo*, suggesting that a potential role of ICA regulating stem cell migration for endogenous repair of IVDD.

Research shows that hypoxic preconditioning can upregulate the migration and proliferation of BMSCs in degenerated IVD and accelerate the effect of BMSC implantation in the treatment of IVDD [28]. ICA can improve metabolic dysfunction of long bone and also prevent bone loss caused by estrogen deficiency [29, 30]. ICA induces the growth and proliferation of neural stem cells by regulating mRNA and protein expression of cell cycle genes, including p21 and cyclin D1 [16]. ICA induces chondrogenic differentiation of BMSCs in self-assembling peptide nanofiber hydrogel scaffolds [31]. Simultaneously, CCK8, EdU and phalloidin staining, transwell assays showed that 1 μM ICA obtained a better proliferation, migration, and bone formation abilities for ISN-SCs, demonstrating that ICA had the ability to promote ISN-SCs migration and bone differentiation in this study.

The extracellular matrix of intervertebral disc mainly include type I and II collagen [32]. This collagen structure is also destroyed by proteolysis due to the maladjustment of collagenase activity, leading to the weakening of mechanical strength and the formation of non-enzymatic cross-linking between reducing sugars and the basic amino acids of collagen [33, 34]. With the degeneration of IVD and dehydration, most of the matrix develops with the decrease of elastin, aggrecan, and glycosaminoglycan, the increase of collagen and collagen cross-linking, and the fracture of aggrecan [35]. In addition, SOX9 is the transcription factor of aggrecan, which also plays a role in IVDD [36]. Simultaneously, our results showed that ICA treatment could reverse the mRNA and protein expression levels of Aggrecan, SOX9, Collagen I, and Collagen II in IVD tissues of IVDD group, demonstrating the role of ICA in repairing IVDD. Moreover, IGF-1 promotes BMSC migration and proliferation in myocardial infarction [37]. Integrin beta-like 1 enhances hepatocellular carcinoma cell invasion and migration via stimulating the TGF-β expression [38]. SDF-1 enhances BMSC migration [39] and NP cells can secrete CCL-5 that induces MSC chemotaxis [11]. In addition, our results showed that ICA treatment also could reverse the protein and mRNA expression levels of IGF-1, TGF-β, SDF-1, and CCL-5 in IVD tissues of IVDD group, and increase the stem cell expression levels, suggesting that ICA might promote the repair of IVDD by inducing chemokines.

## Conclusions

In brief, ICA, the active component of traditional Chinese medicine Herba epimedii, promoted the migration of ISN-SCs to repair degenerated IVD by increasing the expression of chemotactic cytokines, including IGF-1, TGF-β, SDF-1, and CCL-5.

## Supplementary Information

**Additional file 1.**

## Data Availability

The data obtained in this research are available from the corresponding author on reasonable request.

## Abbreviations

ICA: Icariin
SCNs: Stem cell niches
ISN: Intervertebral disc region
ISN-SCs: Intervertebral disc region-derived stem cells
IVDD: Intervertebral disc degeneration
TGF-β: Transforming growth factor
SDF-1: Stromal cell-derived factor-1
CCL-5: Chemokine C–C motif ligand-5
