# Supplementary figures and images for: Icariin regulates stem cell migration for endogenous repair of intervertebral disc degeneration by increasing the expression of chemotactic cytokines
Source: BMC Complement Med Ther. 2022 Mar 10;22:63. doi: 10.1186/s12906-022-03544-x (PMC8915518; doi:10.1186/s12906-022-03544-x)

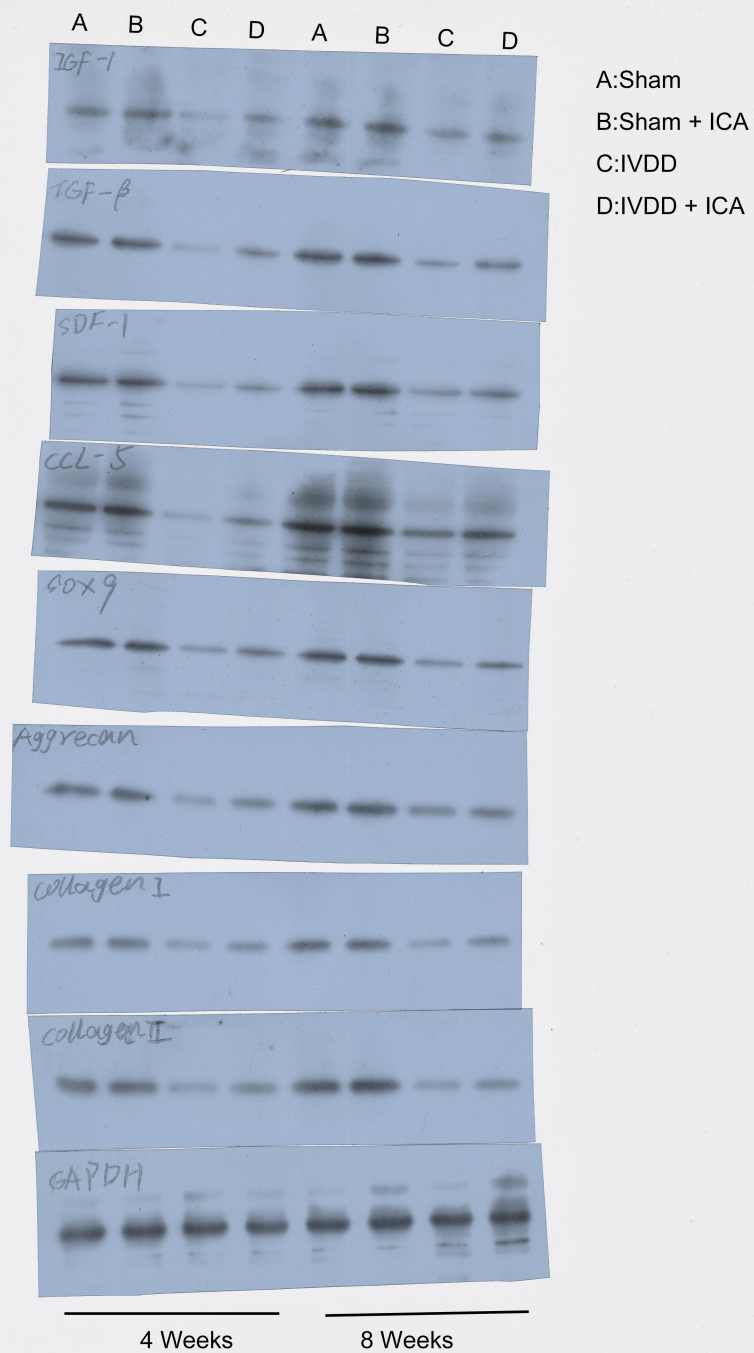

Supplement: Supplementary file 1 — Additional file 1. [file 12906_2022_3544_MOESM1_ESM.pdf]
